# Evaluation of sgRNA Target Sites for CRISPR-Mediated Repression of *TP53*

**DOI:** 10.1371/journal.pone.0113232

**Published:** 2014-11-14

**Authors:** Ingrid E. B. Lawhorn, Joshua P. Ferreira, Clifford L. Wang

**Affiliations:** Department of Chemical Engineering, Stanford University, Stanford, California, United States of America; University College London, United Kingdom

## Abstract

The CRISPR (clustered regularly interspaced short palindromic repeats) platform has been developed as a general method to direct proteins of interest to gene targets. While the native CRISPR system delivers a nuclease that cleaves and potentially mutates target genes, researchers have recently employed catalytically inactive CRISPR-associated 9 nuclease (dCas9) in order to target and repress genes without DNA cleavage or mutagenesis. With the intent of improving repression efficiency in mammalian cells, researchers have also fused dCas9 with a KRAB repressor domain. Here, we evaluated different genomic sgRNA targeting sites for repression of *TP53*. The sites spanned a 200-kb distance, which included the promoter, transcript sequence, and regions flanking the endogenous human *TP53* gene. We showed that repression up to 86% can be achieved with dCas9 alone (i.e., without use of the KRAB domain) by targeting the complex to sites near the *TP53* transcriptional start site. This work demonstrates that efficient transcriptional repression of endogenous human genes can be achieved by the targeted delivery of dCas9. Yet, the efficiency of repression strongly depends on the choice of the sgRNA target site.

## Introduction

CRISPR (clustered regularly interspaced short palindromic repeats) serves as an adaptive immune system for many bacteria and archaea [1], [2]. In the *Streptococcus pyogenes* type II CRISPR system, CRISPR-associated 9 nuclease (Cas9) binds to an RNA complex of trans-acting RNA (tracrRNA) and CRISPR RNA (crRNA), which guides the complex to DNA sequences complementary to the crRNA. This Cas9-RNA complex can then recognize and cleave the DNA of pathogens or other foreign elements [3]–[5]. To develop a general, host-independent method for targeting Cas9 and other recombinant proteins to desired DNA sequences, researchers have devised a platform that utilizes a small guide RNA (sgRNA) consisting of the two RNA elements joined by a short hairpin [5]. Targeting is achieved by the base-pairing between a sequence element encoded by the sgRNA and a desired, target DNA sequence. Any sequence can be targeted as long as it lies upstream of a protospacer adjacent motif (PAM) consisting of the sequence NGG (N represents any nucleotide base) [6], [7]. Thus far, researchers have employed this technology to achieve targeted genomic insertions, deletions, and mutations in bacteria and eukaryotic cells [8]–[14].

More recently, researchers have adapted the CRISPR technology to deliver proteins of interest to targeted DNA sequences for applications beyond genome editing. By inactivating the two endonucleolytic domains of Cas9 to create a catalytically “dead” Cas9 (dCas9) protein [5], researchers have employed the CRISPR gene-targeting platform and conjugated effector domains to achieve transcriptional repression [15]–[18], activation [18]–[21], and genomic loci imaging [22]. The CRISPR-mediated repression of gene expression has been termed CRISPR interference (CRISPRi). With CRISPRi, when dCas9 was co-expressed with sgRNA that was complementary to targeted gene, up to 99.7% transcriptional repression was observed in bacteria [15]. Such repression was achieved by targeting the non-template DNA strand of the gene coding region to block or hinder RNA polymerase transcription elongation [23]. Alternatively, repression has been achieved by targeting sites within a gene promoter region to block RNA polymerase and transcription factor binding for transcription initiation.

In contrast to repression of genes in prokaryotes, recent reports of dCas9-mediated repression in mammalian cells have been more modest, with repression reported to be approximately 50%. To improve on the repression reported in mammalian cells, Gilbert *et al.* fused a Krüppel-associated box (KRAB) transcription repressor domain from Kox1 to the C-terminus of dCas9 and multiple sgRNA target sites against the same gene locus were evaluated [16]. While the KRAB effector domain generated up to 93% repression of a reporter gene expression in human cells, the extent of endogenous gene knockdown varied from gene to gene with, in the best case, up to 80% repression of transferrin receptor CD71 at the protein level [16].

The rules for achieving CRISPR-mediated repression in mammalian systems are still not clearly established. When targeting the transcribed regions of a gene, in bacteria it has been reported that targeting the non-template strand leads to greater repression than when targeting the template strand. Based on this finding, it has been previously recommended that only the non-template strand should be targeted for repression in mammalian cells [23], though to our knowledge this has not been explicitly evaluated outside of bacterial systems. There is evidence that the extent of repression can be greater when targeting DNA sequences close to the transcriptional start site (TSS) in bacteria [15] and human embryonic stem cells [18] than sites in the transcribed region. However, to our knowledge a systematic comparison of promoter-proximal and transcribed region sites has not yet been conducted in mammalian cells. Last, while the KRAB effector domain has been to shown to be more effective than dCas9 alone in a select number of genes [16], it is unclear whether the use of the KRAB effector domain is required to achieve strong endogenous repression.

A better understanding of how the choice of dCas9 target sites affects repression of mammalian gene expression would help those who seek to control gene expression for research or synthetic applications [24]. To address these issues, we evaluated the effect of dCas9-mediated transcriptional repression by using sgRNAs complementary to a variety of target sites covering a 200 kb distance: these sites include the promoter, transcribed sequence and regions adjacent to the endogenous *TP53* gene locus on both the template and non-template DNA strands. p53, the gene product of *TP53*, is a transcription factor whose activation by DNA damage or other cellular stresses results in responses that promote cell cycle arrest, apoptosis, or other tumor suppressing processes [25] and has, previously and presently, served as a well-studied model gene for the purpose of studying gene repression [26], [27]. We also evaluated long-distance repression achieved by dCas9-KRAB binding in regions flanking the *TP53* gene locus. Finally, we evaluated whether repression could be achieved by targeting sites surrounding the transcriptional start site in other genes.

## Materials and Methods

### Vector design

Target sequences were designed using the protocol recommended in Larson *et al.* [23]. Briefly, targets of 20–25 nucleotides in length preceding an NGG PAM site were screened for off-target homology using NCBI BLAST and the CRISPR design tool on crispr.mit.edu. Targets were designed to bind to areas of interest within the human *TP53* promoter as well as to both the template and non-template strands of *TP53* exons 4, 7, and 10. Because the U6 promoter was used for sgRNA expression, all target sequences without a “G” nucleotide in the leading position had one appended to the 5′ end of the target sequence to facilitate expression. Additionally, sgRNA sequences were designed to target sites approximately every 10 kilobases away from the transcriptional start site of *TP53* in both the upstream and downstream directions. GFP targets against the non-template strand were created based on the sequences published in Gilbert *et al.* (GFP1) [16] and Mali *et al.* (GFP2) [10]. Additional targets were designed to bind to locations 20 to 50 base pairs upstream or 100–120 base pairs downstream of the transcriptional start site (+1) as identified by the DataBase of Transcriptional Start Sites [28], [29] for various genes of interest in HEK 293 cells (Sequence Read Archive Accession No. SRA003625) [30]. Scramble1 and Scramble2 “target” controls were designed to have low homology to the human or murine genome and are used as a non-targeting control to normalize gene expression. The sgRNA sequences are summarized in Tables S1, S2, and S3.

Annealed oligonucleotides containing the sgRNA sequences were ligated into the plasmid pRSET B-U6 sgRNA-term that was digested with BpiI (ThermoScientific, Pittsburgh, PA, USA). This step results in a RNA polymerase III U6 promoter-driven sgRNA expression cassette consisting of a 20–25 nucleotide domain complementary to a target DNA region, a Cas9-binding hairpin, and a transcription terminator. The U6-sgRNA expression construct was then digested with PspOMI and NotI-HF (New England BioLabs, Ipswich, MA, USA) and ligated into pCru5-1.2.1-BFP-IRES-Blast that was digested with NotI-HF to create pCru5-BFP-IRES-Blast-U6 sgRNA. Mammalian codon-optimized *Streptococcus pyogenes* dCas9 sequence with nuclease-inactivating D10A and H840A substitutions [5] (Addgene plasmid 44246) was fused to three C-terminal NLS (DPKKKRKV) sequences alone or with the KRAB domain sequence. The entire construct was then inserted into pCru5-1.2-mEGFP-IRES-mCherry-F2A-Puro [31] digested with SphI-HF and NsiI (New England BioLabs) to form pCru5-dCas9-3xNLS-IRES-mCherry-F2A-Puro or its variant with the KRAB domain.

### Cell culture and transfection

HEK 293 [32] and HEK 293T [33] cells were cultured in DMEM with 10% FBS. Media was supplemented with 1 mM glutamine, 100 U/mL penicillin, and 100 µg/mL streptomycin.

HEK 293 and HEK 293T cells were transfected using polyethylenimine (PEI) transfection. Cells plated in 6-well plates were transfected with 2 µg of the dCas9 expression plasmid and 1.2 µg of the sgRNA expression plasmid per well which can be scaled by surface area for larger or smaller plates. Cells transfected with pSuper or pSuper-p53 plasmids were co-transfected with 0.5 µg pCru5-1.2-GFP-IRES-mCherry-F2A-Puro or pEGFP-N3 for selection or sorting purposes. Multiplexed transfection mixes contained equal amounts of all sgRNA expression plasmids used. 1 day post-transfection, cells were split 50% and selected with 2 µg/mL puromycin.

HEK 293 cells were transduced with retrovirus for stable expression of destabilized GFP. Retrovirus was produced by co-transfecting a pCru5 plasmid and pCL-Ampho into HEK 293T cells using calcium phosphate precipitation. One pCru5 plasmid had a long terminal repeat (LTR) containing a promoter that drives transcription of the neomycin resistance gene and an additional mutated EF-1α promoter (EF-T05) [34] driving expression of destabilized monomeric green fluorescent protein (GFP). The other pCru5 plasmid expressed GFP followed by an internal ribosome entry site (IRES) and a neomycin resistance gene directly off the LTR with a translation initiation sequence variant (1.2) [31]. Virus-containing supernatant was harvested and used to transduce HEK 293 cells. Virus was titered so that transduced cells received a single copy of the vectors. Polybrene (hexadimethrine bromide) was added to cultures at a concentration of 8 µg/mL. 24–48 h post-infection, cells were selected and maintained with 400 µg/mL neomycin.

### Flow cytometry

Three days after antibiotic selection, cells were trypsinized, washed once with cold PBS, and resuspended in 1% FBS in PBS. For measurement of GFP knockdown, cells were analyzed on a LSRII (BD Biosciences, San Jose, CA, USA) and gated for mCherry- and TagBFP-positive viable singlet cells. GFP fluorescence on the FITC-A filter was recorded and GFP expression relative to the Scramble1 control was analyzed using FlowJo software (Tree Star, Ashland, OR, USA). For indicated qRT-PCR experiments, samples were sorted on a FACSAria II (BD Biosciences) for TagBFP-positive or GFP-positive viable singlet cells on the Pacific Blue-A filter using the 85 µM nozzle. At least 400,000 cells were sorted per sample.

### Quantitative RT-PCR

Cell samples were trypsinized, washed once with cold PBS, and resuspended in RLT buffer supplemented with β-mercaptoethanol before freezing at −80°C for up to a week. Total RNA was isolated using the RNeasy Mini Kit (Qiagen, Valencia, CA, USA) using the manufacturer's directions. RNA was converted to cDNA using the High-Capacity cDNA Reverse Transcription Kit with random primers using the manufacturer's directions (Applied Biosystems, Foster City, CA, USA). All quantitative PCR reactions were done at 15 µL volume using SYBR Green PCR Master Mix (Applied Biosystems). Each independent biological replicate was plated in triplicate and run on the StepOnePlus thermal cycler (Applied Biosystems). Table S4 lists the primer sequences used to measure expression of *TP53* (GenBank Accession No. NM_000546), *WRAP53α* isoform (GenBank Accession No. NM_001143991), *WRAP53* all isoforms (GenBank Accession No. NM_018081), *GAPDH* (GenBank Accession No. NM_001256799), *MAPK1* (GenBank Accession No. NM_138957), *MAPK14* (GenBank Accession No. NM_001315), *PPIB* (GenBank Accession No. NM_000942), and *RB1* (GenBank Accession No. NM_000321).

Expression levels of housekeeping gene *TIMM17B* (GenBank Accession No. NM_001167947) were used for normalization of human gene expression. Relative quantity was calculated by the comparative C_T_ method (ΔΔCt) using cells transfected with the dCas9 construct and a Scramble sgRNA construct as the control. All reported levels of repression are relative to a Scramble control. Standardization between three independent biological replicates was performed via log transformation, mean centering, and autoscaling described by Willems *et al.* [35]. Relative expression levels from sorted experiments were correlated to their respective target's nucleotide annealing temperature using a linear regression for the target sequence (Figure S4).

### Immunoblotting

To quantify p53 abundance, immunoblotting was performed using standard protocols. Endogenous p53 level was detected in lysate of transfected HEK 293T cells using a mouse anti-p53 primary antibody (1C12, #2524, Cell Signaling Technology, Danvers, MA, USA) and a goat anti-mouse IgG antibody conjugated to horseradish peroxidase (HRP). Loading control GAPDH was detected using a rabbit primary antibody conjugated to HRP (14C10, #3683 Cell Signaling Technology, Danvers, MA, USA). HRP activity was measured with the ECL Plus Western Blotting Detection Kit (#RPN2132, GE Healthcare, Piscataway, NJ, USA).

## Results

Our goal was to investigate how the location of the sgRNA target site within a gene locus affects dCas9-mediated repression in human cells. We constructed a vector that expressed a human codon-optimized dCas9 protein with three nuclear localization signals (3xNLS). The vector also utilized an internal ribosome entry site (IRES) that allowed bicistronic expression of the dCas9 with mCherry-2A-Puro, which encoded both the mCherry red fluorescent protein and a puromycin resistance gene (Figure S1A). To evaluate the efficiency of the KRAB effector domain, a version was also created with KRAB fused to the C terminus of the dCas9-3xNLS fusion (Figure S1A). Each sgRNA was expressed from a RNA polymerase III U6 promoter downstream of a cassette expressing blue fluorescent protein (BFP) and a blasticidin resistance gene (Figure S1A). To test the efficacy of our dCas9 fusions, we co-transfected the dCas9 constructs and individual sgRNAs in HEK 293 cells stably expressing green fluorescent protein (GFP). We observed reduction of GFP fluorescence over the non-targeted Scramble1 control in cells expressing a sgRNA targeting GFP and either dCas9 (37% repression) or dCas9-KRAB (60% repression) (Figure S2). As observed previously [16], dCas9-KRAB generated greater GFP repression than dCas9 alone.

In this study, we were interested in evaluating the repression of endogenous genes. Having established that our constructs could repress ectopic GFP expression, we next evaluated the effectiveness of dCas9 and dCas9-KRAB in repressing human *TP53* mRNA expression. To evaluate the effect of target site position on repression, we tested sgRNAs that targeted different sites at or near the *TP53* gene and measured *TP53* mRNA levels by quantitative real-time PCR (qRT-PCR). We hypothesized that the KRAB fusion may have long-distance repressive effects on gene expression since KRAB-mediated promoter silencing has been reported over distances of several tens of kilobases away from the KRAB binding site [36]. To evaluate this hypothesis, we designed sgRNAs to target sites up to 100,000 bp upstream and downstream of the *TP53* transcriptional start site (TSS) in approximately 10,000 bp increments (Figure S1B). Each sgRNA is labeled with a number representing the distance (bp) from the transcriptional start site (“K”, one thousand bp) and a “−” or “+” representing upstream or downstream of +1. HEK 293T cells were co-transfected with a dCas9 or dCas9-KRAB expression construct and each sgRNA expression construct, selected with puromycin, and sorted for BFP positive cells. Of the 21 sgRNAs tested, the only appreciable repression of mRNA occurred at a site approximately 36 bp upstream from the *TP53* TSS (85% for dCas9; 90% for dCas9-KRAB; −T36, Figure S1C and Figure S1D). Furthermore, there was little observable additional *TP53* repression with the dCas9-KRAB fusion in comparison to repression with dCas9. Since using multiple sgRNAs has been successful in CRISPR-directed gene activation [19], [20], we tested the effect of using multiple sgRNAs for repression by co-transfecting dCas9 or dCas9-KRAB with various combinations of sgRNAs targeting: 10 sites spanning 100,000 bp upstream of *TP53* (−T100K to −T10K), three sites spanning 30,000 bp upstream of *TP53* (−T30K to −T10K), 10 sites spanning 100,000 bp downstream of *TP53* (+T10K to +T100K), three sites spanning 30,000 bp downstream of *TP53* (+T10K to +T30K), and three sites flanking the *TP53* TSS (−T36 +T27 +T110). Again, there was no added transcriptional repression with dCas9-KRAB directed binding as compared to dCas9 (Figure S1E). Moreover, we observed similar repression of *TP53* with the simultaneous targeting of three sites (−T36 +T27 +T100) as with the targeting of the single −T36 site (Figure S1D). This is in contrast to the previously-observed synergistic increase in gene activation induced by use of multiple sgRNA binding sites in conjunction with a dCas9 fused to aVP64 transcription activation domain [20], tetramer of VP16 domains, each capable of recruiting transcriptional machinery to promoter proximal regions. We postulate that this synergy in activation occurred because activation by VP64 can act over long distances [37] thus allowing multiple targeted sites to act together from various distances. In contrast, the repression that we observed may be due to a TSS-proximal mechanism, where additional dCas9 repression factors cannot cooperate significantly to improve the degree of repression. Our results also suggest that fusing KRAB to dCas9 does not significantly improve *TP53* transcriptional repression and that targeting multiple sites concurrently does not necessarily improve the efficiency of repression. However, we cannot rule out the possibility that KRAB may increase repression when targeted to other genes or using alternative sites.

**Figure 1:**
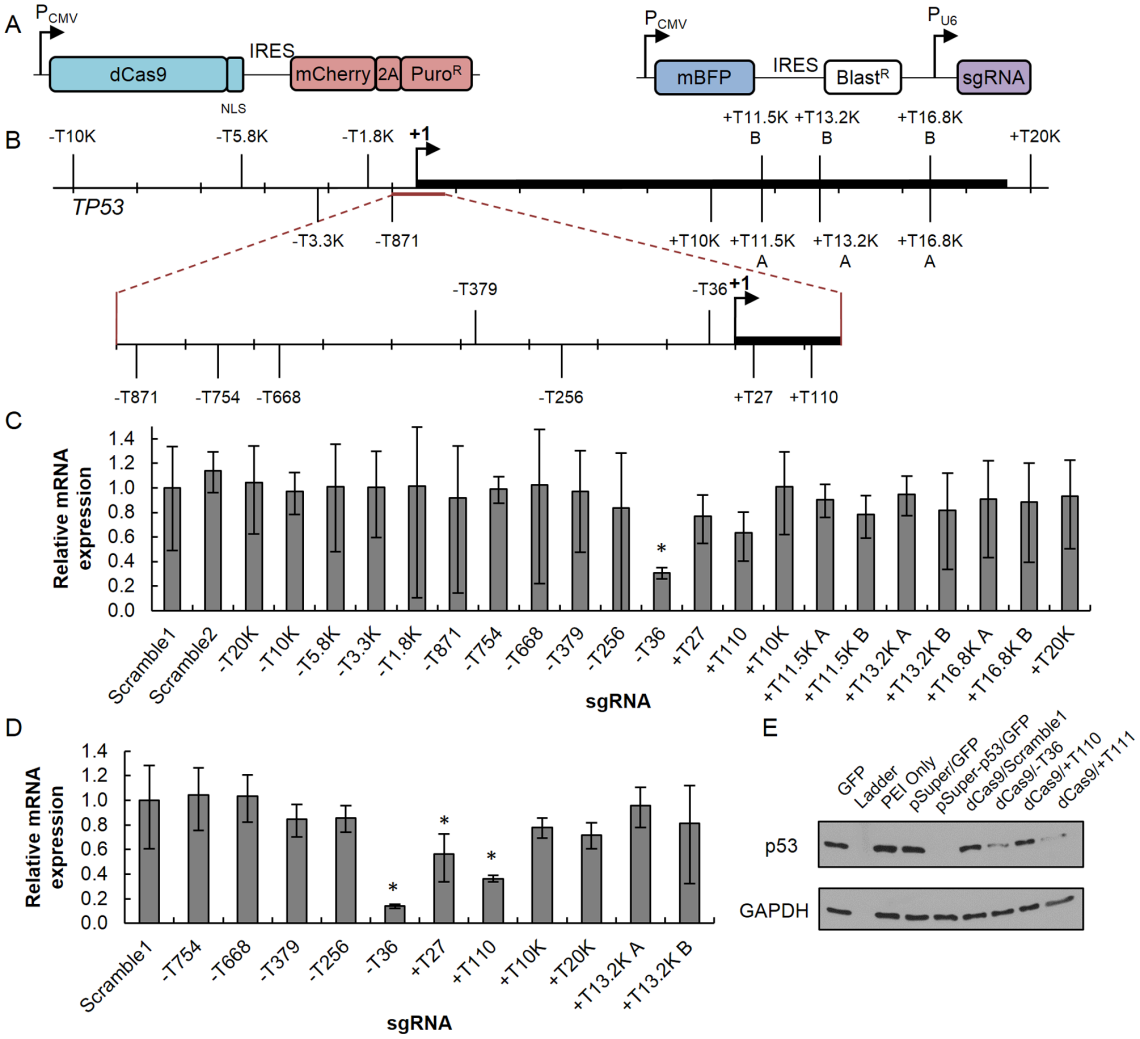
Repression of *TP53* by targeting dCas9 to the transcriptional start site. (A) HEK 293T cells were co-transfected with dCas9 (left) and sgRNA (right) expression plasmids. Codon-optimized dCas9 was fused to three copies of nuclear localization signal (NLS) and was co-expressed with mCherry fluorescent protein. sgRNA plasmid expresses mBFP and sgRNA off separate promoters. P_CMV_, CMV promoter; 2A, ribosomal slippage site; Puro^R^, puromycin resistance gene; IRES, internal ribosome entry site; mBFP, TagBFP fluorescent protein; Blast^R^, blasticidin resistance gene; P_U6_, U6 promoter. (B) Locations of sgRNA binding sites in the *TP53* promoter and transcribed region (thick line). Each sgRNA is numbered by the distance (bp) from the transcriptional start site. “−”, upstream of +1; “+”, downstream of +1; “K”, one thousand bp; +1, transcriptional start site. Labeled sites above and below the transcribed region indicate sgRNAs targeting the template or non-template DNA strands, respectively. (C, D) Relative expression of *TP53* mRNA in cells co-transfected with dCas9 and indicated sgRNA constructs. After three days, cells were (C) directly analyzed by qRT-PCR or (D) sorted for BFP-positive cells, then analyzed by qRT-PCR. Results were normalized, linearly rescaled, and calculated for mean fold change (*n* = 3)±95% confidence interval, relative to Scramble1 negative control sgRNA. *P<0.01 compared to non-targeted sgRNA control by paired, one-sided t-test. (E) Immunoblot from HEK 293T cell lysate three days post-transfection with shRNA against *TP53*, dCas9 and sgRNAs against *TP53* (e.g., dCas9/−T36), dCas9 and non-targeted sgRNA (dCas9/Scramble1), other control constructs (GFP expression vector, which served as non-specific vector control, or pSuper without any shRNA encoded), or mock (PEI only). See Figure 2E for location of sgRNA +T111. pSuper constructs were co-transfected with GFP-IRES-mCherry-2A-Puro expression constructs for selection purposes and experimental consistency (pSuper/GFP, pSuper-p53/GFP). Protein ladder (lane 2 from left) is not visible. See Figure S5 for uncropped immunoblots.

Since dCas9-KRAB had little added benefit over dCas9 for repression of *TP53* and there was significant repression when targeting sites near the TSS, we further explored the positional effect of targeting dCas9 to sites closer to the *TP53* gene itself. 21 sgRNAs were designed to target sequences on the template and non-template strands of the promoter and transcribed region of *TP53* (Table S1 and Figure 1A and B). In bacteria, only sgRNAs binding to the non-template strand could enable repression of a fluorescent protein [15] although that finding has not been replicated in mammalian cells. The sgRNAs were co-expressed with dCas9 in transfected and puromycin-selected HEK 293T cells and directly analyzed by qRT-PCR. As before, the −T36 target site was shown to significantly repress *TP53* expression at the mRNA level (*P*<0.01, Figure 1C) as compared to cells expressing either non-targeted control. Because transfection efficiency varied, we opted to sort for BFP-positive cells after transfection and puromycin selection to enrich for cells expressing both CRISPR constructs. Of the subset of sgRNAs tested, three repressed *TP53* transcriptional expression (Figure 1D), all located within a 200-bp region of the TSS. sgRNA −T36 induced approximately 86% *TP53* mRNA repression, a level comparable to shRNA knock-down with pSuper-p53 construct [26] (Figure S3C). Repression using this target site was also consistently observed at the protein level in unsorted, selected cells (Figure 1E) for three independent experiments. However, when targeting the +T110 and +T111 sites and analyzing protein levels by immunoblotting, not only were the magnitudes of repression lower than −T36 but they were also less reproducible over those same experiments. Additionally, the targeting of sites +T27 and +T110 repressed *TP53* transcription expression. We did not observe significant repression when targeting the template or non-template strands of DNA in the transcribed region. It should be noted that first exon of *TP53* overlaps with exon 1α of *WRAP53* [38], [39] which is the first exon of the *WRAP53α* isoform—a known anti-sense transcript of *TP53* (Figure S3A). We observed repression of the *WRAP53α* isoform in cells transfected with dCas9 and the −T36 sgRNA but no similar repression of total *WRAP53* expression from all three isoforms (Figure S3B); we did not see a similar effect with pSuper-p53 shRNA transfection (Figure S3C). Finally, we note that for the many sgRNAs in our study that did not generate appreciable repression of TP53, we cannot rule out that these sgRNAs were somehow incapable of mediating the binding of dCas9 to the DNA.

**Figure 2:**
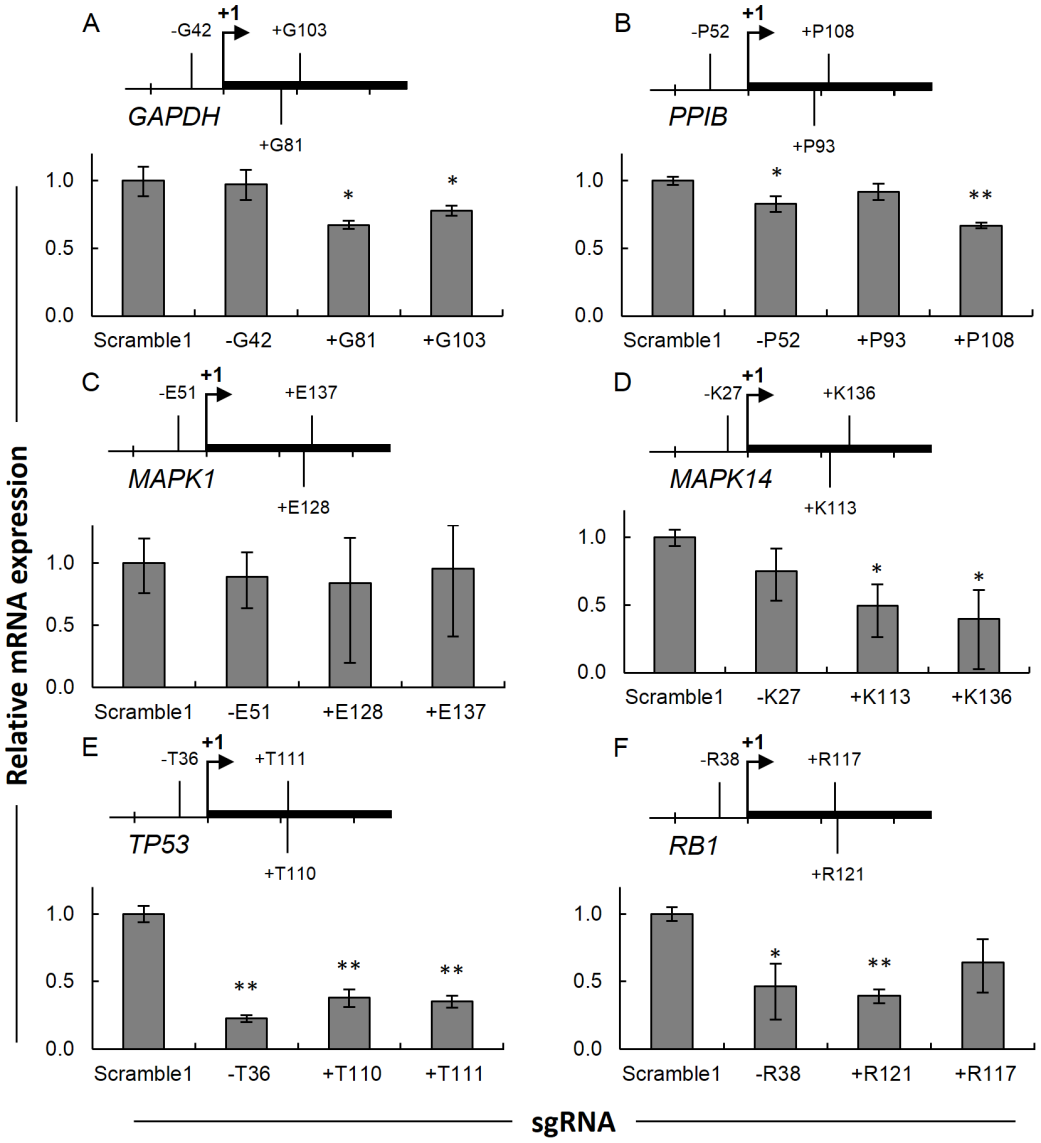
Evaluation of dCas9 targeting to the TSS of various genes. Diagram of sgRNA binding sites and plots of mRNA repression in HEK 293T by dCas9 and sgRNAs targeting (A) *GAPDH*, (B) *PPIB*, (C) *MAPK1*, (D) *MAPK14*, (E) *TP53*, and (F) *RB1*. Each sgRNA is numbered by the distance from the transcriptional start site. “−”, upstream of +1; “+”, downstream of +1; +1, transcriptional start site. Labeled sites above and below the transcribed region indicate sgRNAs targeting the template or non-template DNA strands, respectively. Relative expression of each mRNA in HEK 293T cells co-transfected with dCas9 and sgRNA constructs. After three days, cells transfected with dCas9 and indicated sgRNA constructs were sorted for BFP-positive cells, analyzed by qRT-PCR. Results were normalized, linearly rescaled, and calculated for mean fold change (*n* = 3)±95% confidence interval, relative to Scramble1 negative control sgRNA. *P<0.02, **P<0.002 compared to Scramble1 sgRNA control by paired, one-sided t-test.

Observing that target sites −T36 and +T110 were effective at repressing *TP53* transcription, we next designed sgRNAs to target sites at similar distances from the TSS for five additional human genes: *GAPDH*, *PPIB*, *MAPK1*, *MAPK14*, and *RB1* (Figure 2). For each gene, dCas9 was targeted to one site 27 to 52 bp upstream of the TSS on the non-template DNA strand and two sites 81 to 136 bp downstream of TSS on the both the non-template and template DNA strands (Table S3). While it is certainly possible that the KRAB fusion could be effective for repression of these other genes, because we found the dCas9 alone was sufficient for repression of *TP53*, we chose to investigate the whether dCas9 alone could also be effective in repressing other genes. For four of the five genes, modest transcriptional repression was achieved, including over 50% repression in *RB1* with target +R121 (Figure 2F). To explain the variation in repression efficiency, we hypothesized that the annealing temperature of the target base pair sequence may correlate with the efficiency of repression. Analysis of this parameter and the relative expressions for each target examined in triplicate revealed no correlation between the sgRNA target annealing temperature and mRNA expression (Figure S4). These results indicate modest transcriptional repression can be achieved in a variety of genes by targeting the region proximal to the TSS although there may be additional factors limiting the extent of that knockdown.

## Discussion

Prior reports [16], [23] recommended targeting either the promoter-proximal region or the transcribed region on the non-template strand for optimal endogenous dCas9 repression in human cells. In the case of *TP53*, while we were unable to achieve significant repression by targeting various sites on either the template or non-template strand of the transcribed region, we were able to repress transcription by targeting sites near or close to the TSS. We report up to 86% repression of *TP53* transcription by using sgRNAs −T36 and +T110, targeting sites 36 bp upstream and 110 bp downstream of the transcriptional start site. Interestingly, the −T36 sgRNA binds to a site that overlaps with previously-reported Myc/Max, USF, and E2F1 binding motifs while +T110 binds to a site overlapping with a Pax binding motif [40]. The −T36 sgRNA is also within 40 bp of the transcription start site, which is the approximate DNA footprint of RNA polymerase II [41]. In support of the notion the dCas9 can interfere with transcription at the DNA level,, recent chromatin immunoprecipitation (ChIP) data by others indicate that sgRNA-mediated dCas9 binding at the transcriptional enhancer of *Nanog* interferes with the binding of several transcription factors in mouse ESCs [42].The CRISPR-mediated repression that we observed is similar to what we were able to achieve using a *TP53* shRNA construct [26] and similar to levels of repression Gilbert *et al.* achieved by targeting sites downstream of the TSS of endogenous *CD71* and *CXCR4* genes [16]. We did not find a clear association between target proximity to the TSS and transcriptional repression, but we were able to achieve modest but significant repression in an additional four of five examined genes.

Interference with transcription initiation provides an alternative to post-transcriptional regulation via RNAi and allows for evaluation of repression at the transcriptional-level. In contrast to gene editing with RNA-directed Cas9, dCas9-mediated repression allows for a reduction in gene expression without modification to the genomic sequence. Thus with dCas9-mediated repression, it should be possible to engineer conditional expression system where repression can be switched “on” or “off” reversibly by other cellular cues or experimental conditions (e.g., addition of a small molecule inducer). In addition, it appears that, depending on the targeted gene, similar levels of transcriptional repression can be achieved without the use of a KRAB effector domain, which, for *TP53* transcription, appeared to have little effect. It is important to note potential limitations in the dCas9-initiated repression. Access of the dCas9-RNA complexes to the target site may be limited or even obstructed by the structure and local environment of the chromatin, epigenetic features, or the presence of regulatory elements [43], [44] and thus affect efficiency of repression. Additionally, even if a sequence has few if any off-target binding sites [44]–[48], some promoter regions may overlap with other genes. We observed concomitant repression of *WRAP53α* isoform mRNA levels (Figure S3B) when targeting *TP53*'s TSS.

To summarize, our results demonstrate significant CRISPRi repression of *TP53* mRNA levels without use of a KRAB effector domain by targeting the TSS. Modest repression was also observed in four of five genes targeted at the TSS. Our study demonstrates that target site efficiencies can vary greatly. When one desires to utilize CRISPRi to repress genes in mammalian cells, one should evaluate a range of sgRNA target sites, including those near or at the transcriptional start site. However, the targeting of the transcriptional start site is not guaranteed to be effective for every gene and thus will need to be evaluated on a gene-by-gene basis. We propose that CRISPRi with dCas9 alone may prove useful for functional mapping of transcription factor motifs and enhancer elements via transcription initiation blocking without the potential additional repressive effect of a KRAB domain.

## Supporting Information

Figure S1 **Evaluation of CRISPR-mediated repression using dCas9 and dCas9 fused to a KRAB repressor domain.** (A) HEK 293T cells were co-transfected with dCas9 or dCas9 fused to KRAB domain (left) and sgRNA (right) expression plasmids. Both codon-optimized dCas9 and dCas9-KRAB were fused to three copies of nuclear localization signal (NLS) and were co-expressed with mCherry fluorescent protein. sgRNA plasmid expresses mBFP and sgRNA off separate promoters. P_CMV_, CMV promoter; 2A, ribosomal slippage site; Puro^R^, puromycin resistance gene; IRES, internal ribosome entry site; mBFP, TagBFP fluorescent protein; Blast^R^, blasticidin resistance gene; P_U6_, U6 promoter. (B) Locations of sgRNA binding sites in the endogenous *TP53* locus. Each sgRNA is numbered by the distance (bp) from the transcriptional start site. “−”, upstream of +1; “+”, downstream of +1; “K”, one thousand bp; +1, transcriptional start site. Labeled sites above and below the transcribed region indicate sgRNAs targeting the template or non-template DNA strands, respectively. (C–E) Relative expression of *TP53* mRNA in cells co-transfected with dCas9 and sgRNA constructs targeting (C) upstream, (D) downstream of the +1 site or (E) in combinations of multiple sgRNA. After three days, cells co-transfected with indicated dCas9 and sgRNA constructs were analyzed by qRT-PCR. Data in (C, D) are fold change relative to Scramble1 or Scramble2 negative control sgRNA ± s.e. of three technical replicates. Data in (E) represents sorted cells and were normalized, linearly rescaled, and calculated for mean fold change (*n* = 3)±95% confidence interval, relative to Scramble1 negative control sgRNA. *P<0.01 compared to non-targeted sgRNA control by paired, one-sided t-test. See also Figure 1B for additional targeted *TP53* sites.
(TIF)

Figure S2 **CRISPR-mediated repression of GFP.** Destabilized GFP (ds1GFP) expression cassette expressed using an (A) LTR (P_LTR_) or (B) mutant EF-1α promoter (P_EF-T05_) was retrovirally transduced into HEK 293 cells and selected with neomycin. LTR, retroviral long-terminal repeat; Neo^R^, neomycin resistance gene; IRES, internal ribosome entry site. Cells were analyzed three days post-co-transfection with dCas9-KRAB and either GFP1 or GFP2 sgRNAs targeting the template strand. GFP fluorescence was measured via flow cytometry after gating for BFP positive cells. Values are arithmetic means of GFP fluorescence ± s.d. (*n* = 3) calculated from geometric means of each sample population and were normalized to dCas9:Scramble1 negative control.
(TIF)

Figure S3 **Target sequence −T36 also leads to a reduction in** ***WRAP53α*** **isoform mRNA.** (A) Locations of sgRNA binding sites in the endogenous *TP53* locus. Each sgRNA is numbered by the distance (bp) from the transcriptional start site. “−”, upstream of +1; “+”, downstream of +1; “K”, one thousand bp; +1, transcriptional start site. Labeled sites above and below the transcribed region indicate sgRNAs targeting the template or non-template DNA strands, respectively. (B, C) Relative expression of *TP53*, *WRAP53α*, and all isoforms of *WRAP53* mRNA in cells co-transfected with (B) dCas9 and sgRNA constructs or (C) shRNA constructs targeting *TP53*. After three days, cells co-transfected with indicated constructs were sorted, analyzed by qRT-PCR. Data are fold change relative to (B) Scramble1 negative control sgRNA or (C) pSuper Control plasmid ± s.e. of three technical replicates.
(TIF)

Figure S4 **Annealing temperature of sgRNA target sequence has minimal to no correlation with reduction in transcriptional expression.** Minimal to no correlation was observed between transcriptional repression relative to Scramble1 control of all individual dCas9 knockdown experiments done in triplicate and the annealing temperature of the sgRNA target. Line represents linear regression of data.
(TIF)

Figure S5 **Reduction of p53 protein in transfected HEK 293T cells.** Uncropped immunoblot (see Figure 1E) containing 15 µg total protein/lane immunostained with (A) p53 antibody and (B) GAPDH antibody from HEK 293T cells transfected as indicated. Protein ladder (lane 2 from left) is not visible.
(TIF)

Table S1 **Target sequences for sites within and flanking human** ***TP53*** **.** The leading G nucleotide required for U6 promoter expression is in bold. The underlined following 19 to 24 nucleotides comprise the target sequence. The PAM site is in gray.
(TIF)

Table S2 **Control target sequences.** The leading G nucleotide required for U6 promoter expression is in bold. The underlined following 19 to 24 nucleotides comprise the target sequence. The PAM site is in gray.
(TIF)

Table S3 **Target sequences within the promoter of various human genes of interest.** The leading G nucleotide required for U6 promoter expression is in bold. The underlined following 19 to 24 nucleotides comprise the target sequence. The PAM site is in gray.
(TIF)

Table S4 **Primer sequences used for quantitative RT-PCR.**
(TIF)
