# Supplementary figures and images for: Evaluation of sgRNA Target Sites for CRISPR-Mediated Repression of TP53
Source: PLoS One. 2014 Nov 14;9(11):e113232. doi: 10.1371/journal.pone.0113232 (PMC4232525; doi:10.1371/journal.pone.0113232)

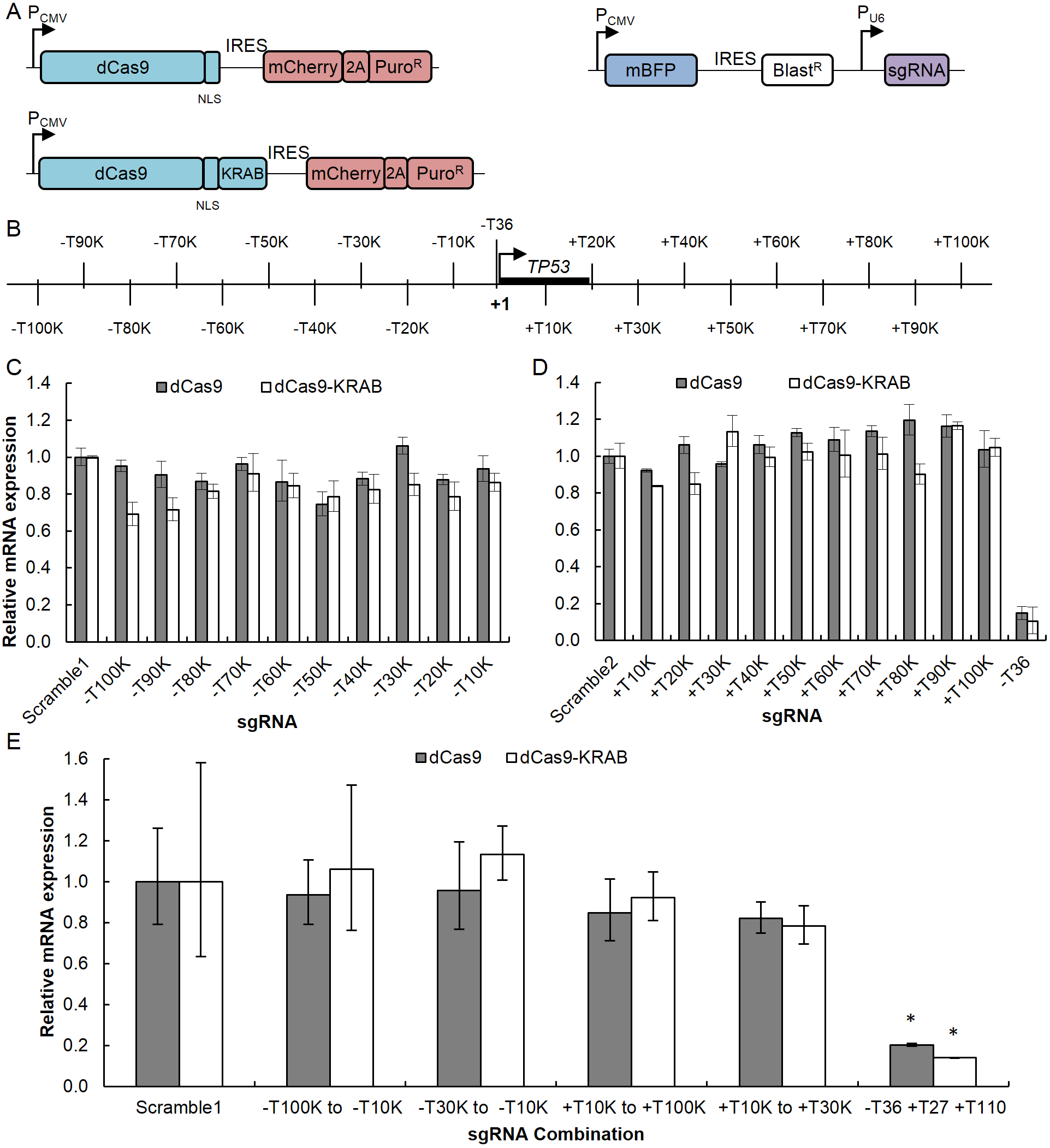

Supplement: Figure S1 — Evaluation of CRISPR-mediated repression using dCas9 and dCas9 fused to a KRAB repressor domain. (A) HEK 293T cells were co-transfected with dCas9 or dCas9 fused to KRAB domain (left) and sgRNA (right) expression plasmids. Both codon-optimized dCas9 and dCas9-KRAB were fused to three copies of nuclear localization signal (NLS) and were co-expressed with mCherry fluorescent protein. sgRNA plasmid expresses mBFP and sgRNA off separate promoters. PCMV, CMV promoter; 2A, ribosomal slippage site; PuroR, puromycin resistance gene; IRES, internal ribosome entry site; mBFP, TagBFP fluorescent protein; BlastR, blasticidin resistance gene; PU6, U6 promoter. (B) Locations of sgRNA binding sites in the endogenous TP53 locus. Each sgRNA is numbered by the distance (bp) from the transcriptional start site. “−”, upstream of +1; “+”, downstream of +1; “K”, one thousand bp; +1, transcriptional start site. Labeled sites above and below the transcribed region indicate sgRNAs targeting the template or non-template DNA strands, respectively. (C–E) Relative expression of TP53 mRNA in cells co-transfected with dCas9 and sgRNA constructs targeting (C) upstream, (D) downstream of the +1 site or (E) in combinations of multiple sgRNA. After three days, cells co-transfected with indicated dCas9 and sgRNA constructs were analyzed by qRT-PCR. Data in (C, D) are fold change relative to Scramble1 or Scramble2 negative control sgRNA ± s.e. of three technical replicates. Data in (E) represents sorted cells and were normalized, linearly rescaled, and calculated for mean fold change (n = 3)±95% confidence interval, relative to Scramble1 negative control sgRNA. *P<0.01 compared to non-targeted sgRNA control by paired, one-sided t-test. See also Figure 1B for additional targeted TP53 sites. (TIF) [file pone.0113232.s001.tif]

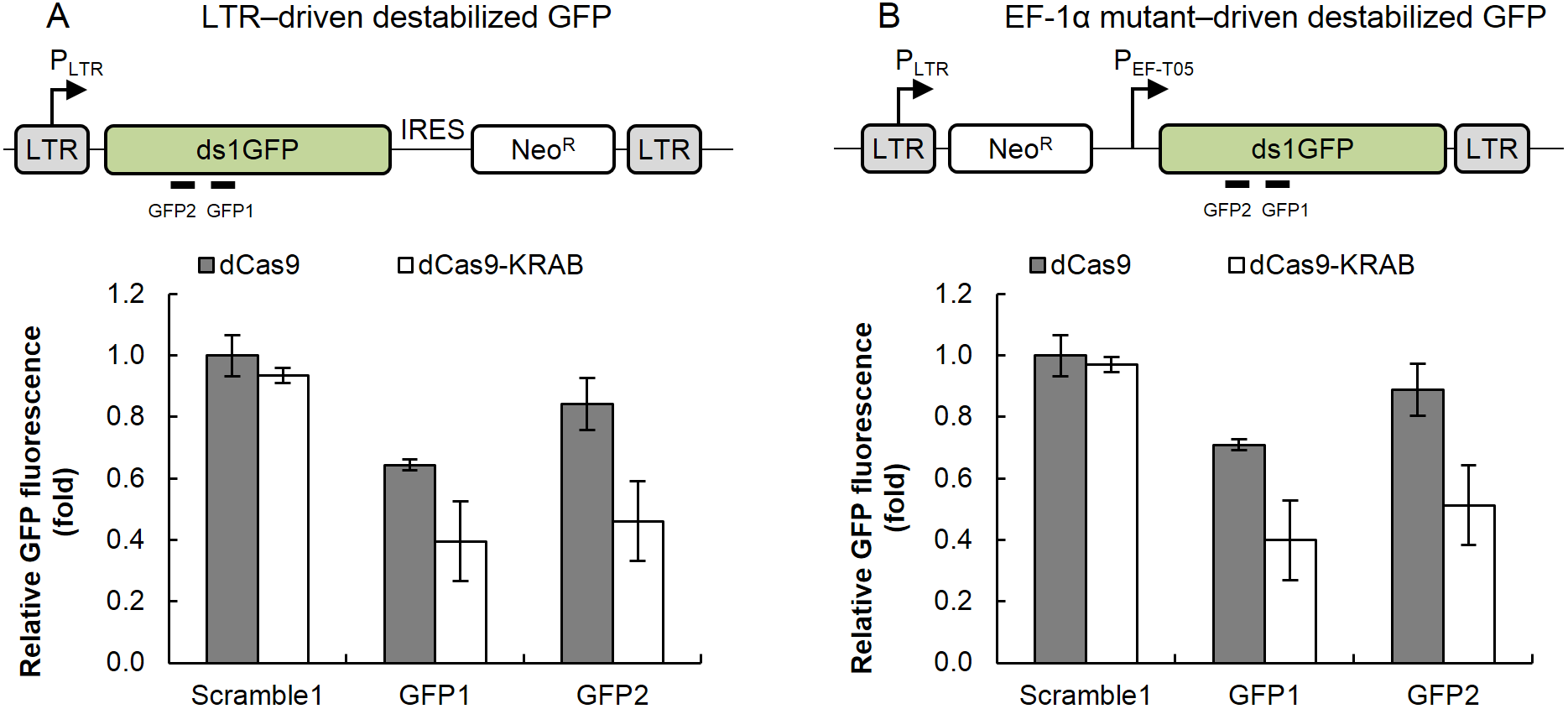

Supplement: Figure S2 — CRISPR-mediated repression of GFP. Destabilized GFP (ds1GFP) expression cassette expressed using an (A) LTR (PLTR) or (B) mutant EF-1α promoter (PEF-T05) was retrovirally transduced into HEK 293 cells and selected with neomycin. LTR, retroviral long-terminal repeat; NeoR, neomycin resistance gene; IRES, internal ribosome entry site. Cells were analyzed three days post-co-transfection with dCas9-KRAB and either GFP1 or GFP2 sgRNAs targeting the template strand. GFP fluorescence was measured via flow cytometry after gating for BFP positive cells. Values are arithmetic means of GFP fluorescence ± s.d. (n = 3) calculated from geometric means of each sample population and were normalized to dCas9:Scramble1 negative control. (TIF) [file pone.0113232.s002.tif]

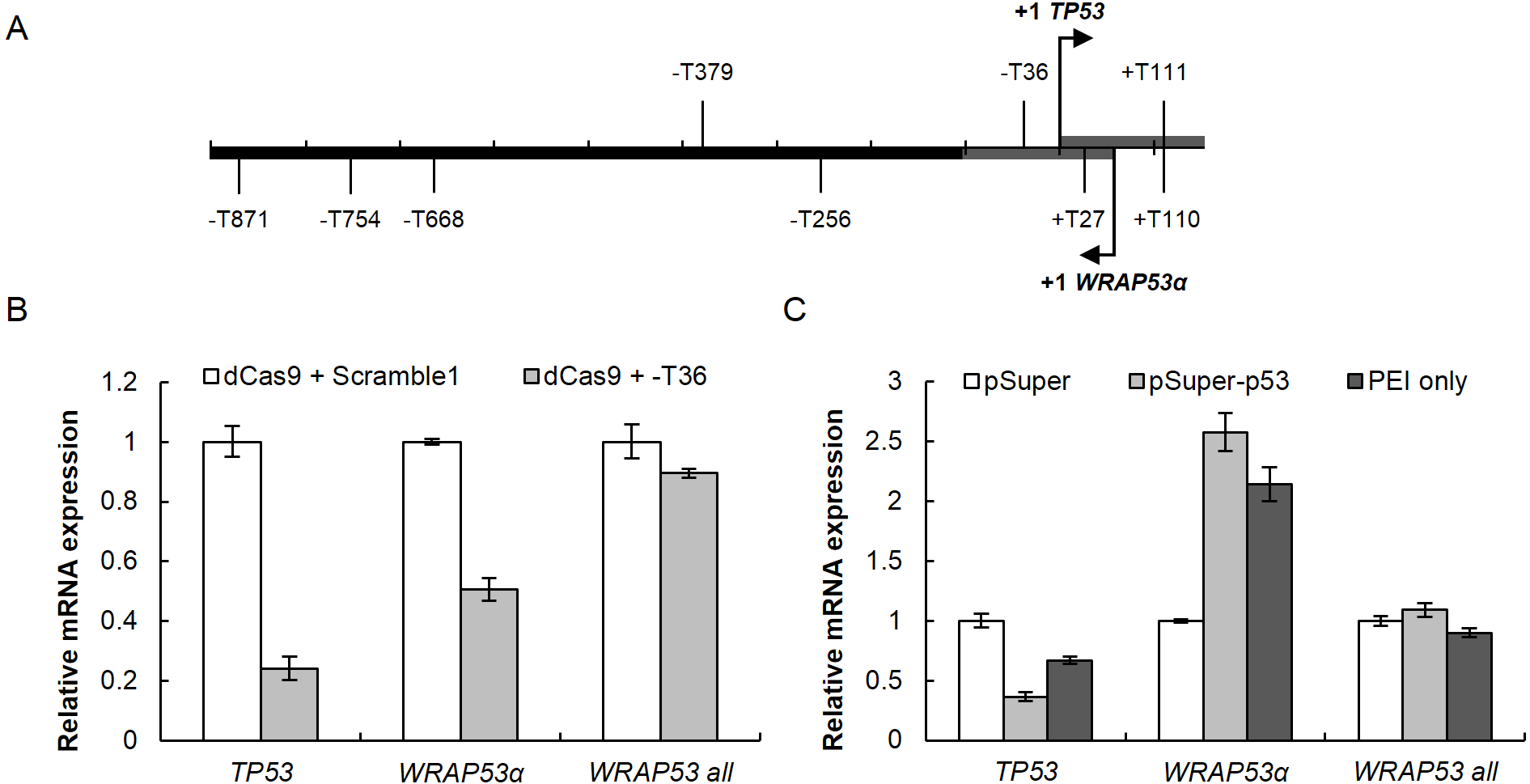

Supplement: Figure S3 — Target sequence −T36 also leads to a reduction in WRAP53α isoform mRNA. (A) Locations of sgRNA binding sites in the endogenous TP53 locus. Each sgRNA is numbered by the distance (bp) from the transcriptional start site. “−”, upstream of +1; “+”, downstream of +1; “K”, one thousand bp; +1, transcriptional start site. Labeled sites above and below the transcribed region indicate sgRNAs targeting the template or non-template DNA strands, respectively. (B, C) Relative expression of TP53, WRAP53α, and all isoforms of WRAP53 mRNA in cells co-transfected with (B) dCas9 and sgRNA constructs or (C) shRNA constructs targeting TP53. After three days, cells co-transfected with indicated constructs were sorted, analyzed by qRT-PCR. Data are fold change relative to (B) Scramble1 negative control sgRNA or (C) pSuper Control plasmid ± s.e. of three technical replicates. (TIF) [file pone.0113232.s003.tif]

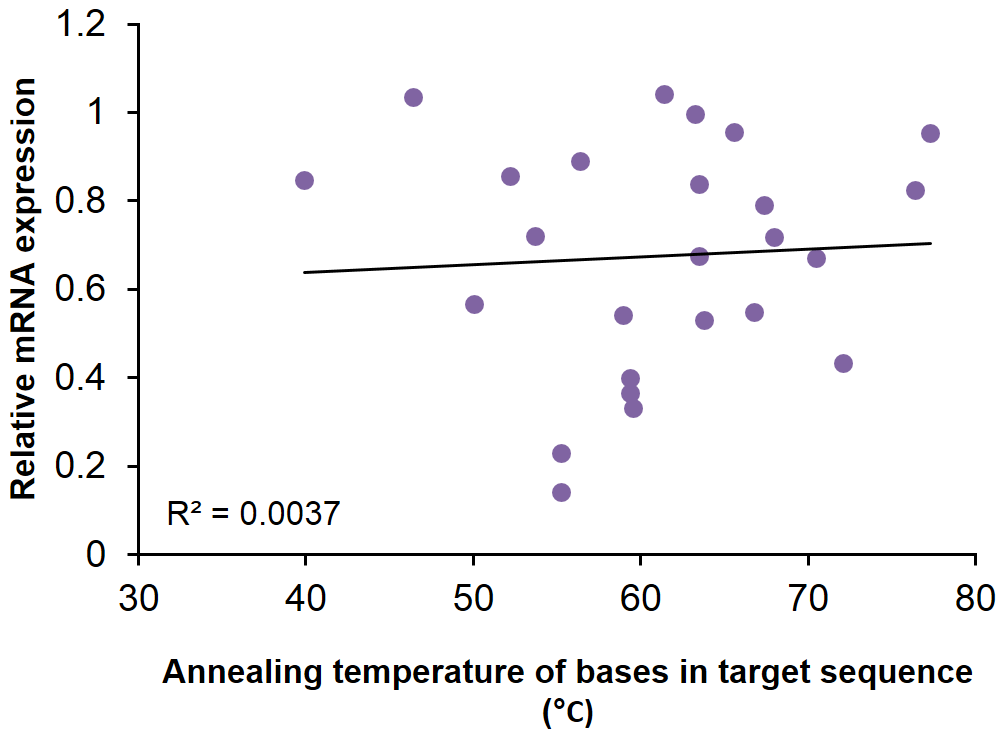

Supplement: Figure S4 — Annealing temperature of sgRNA target sequence has minimal to no correlation with reduction in transcriptional expression. Minimal to no correlation was observed between transcriptional repression relative to Scramble1 control of all individual dCas9 knockdown experiments done in triplicate and the annealing temperature of the sgRNA target. Line represents linear regression of data. (TIF) [file pone.0113232.s004.tif]

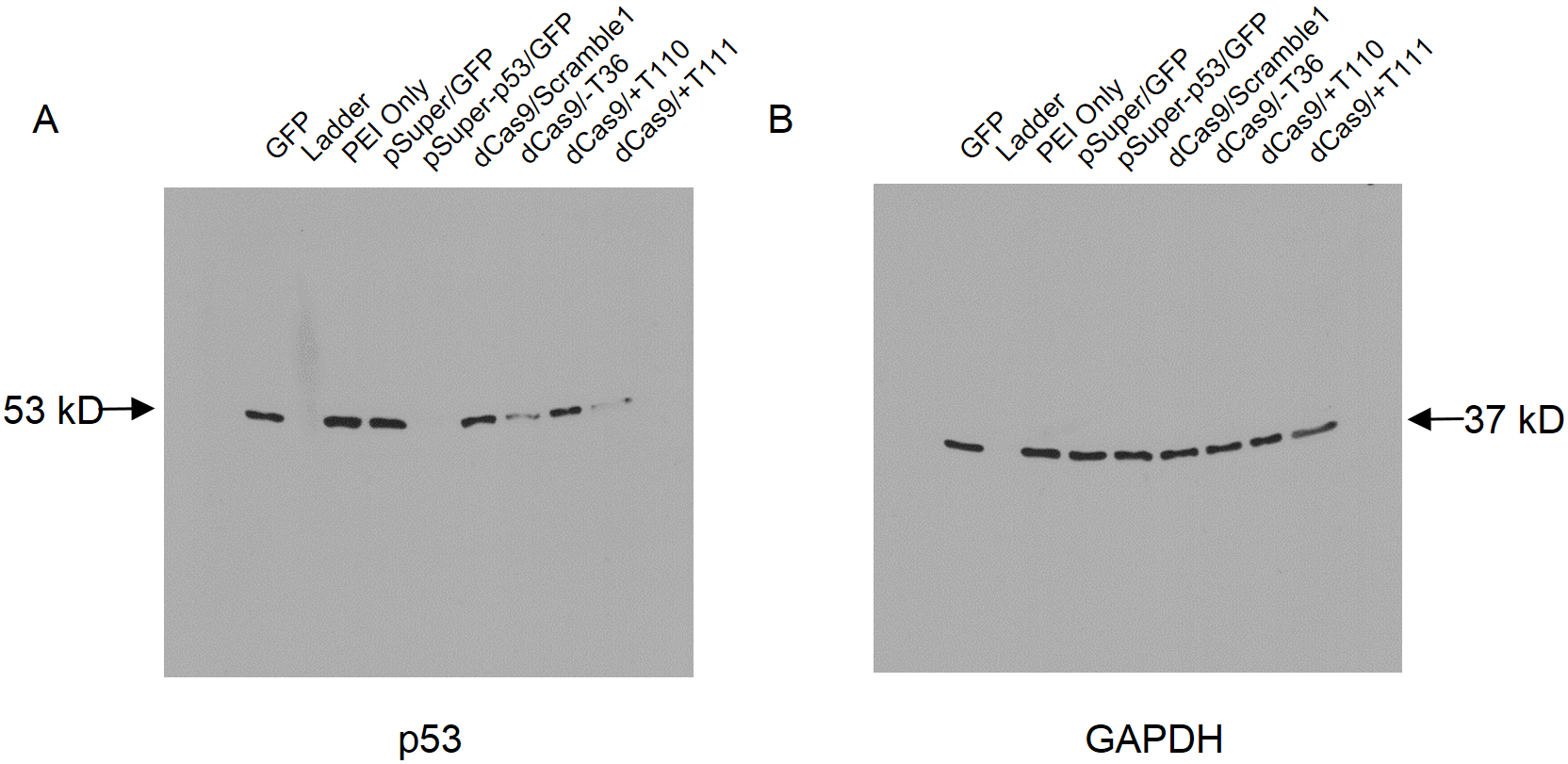

Supplement: Figure S5 — Reduction of p53 protein in transfected HEK 293T cells. Uncropped immunoblot (see Figure 1E) containing 15 µg total protein/lane immunostained with (A) p53 antibody and (B) GAPDH antibody from HEK 293T cells transfected as indicated. Protein ladder (lane 2 from left) is not visible. (TIF) [file pone.0113232.s005.tif]

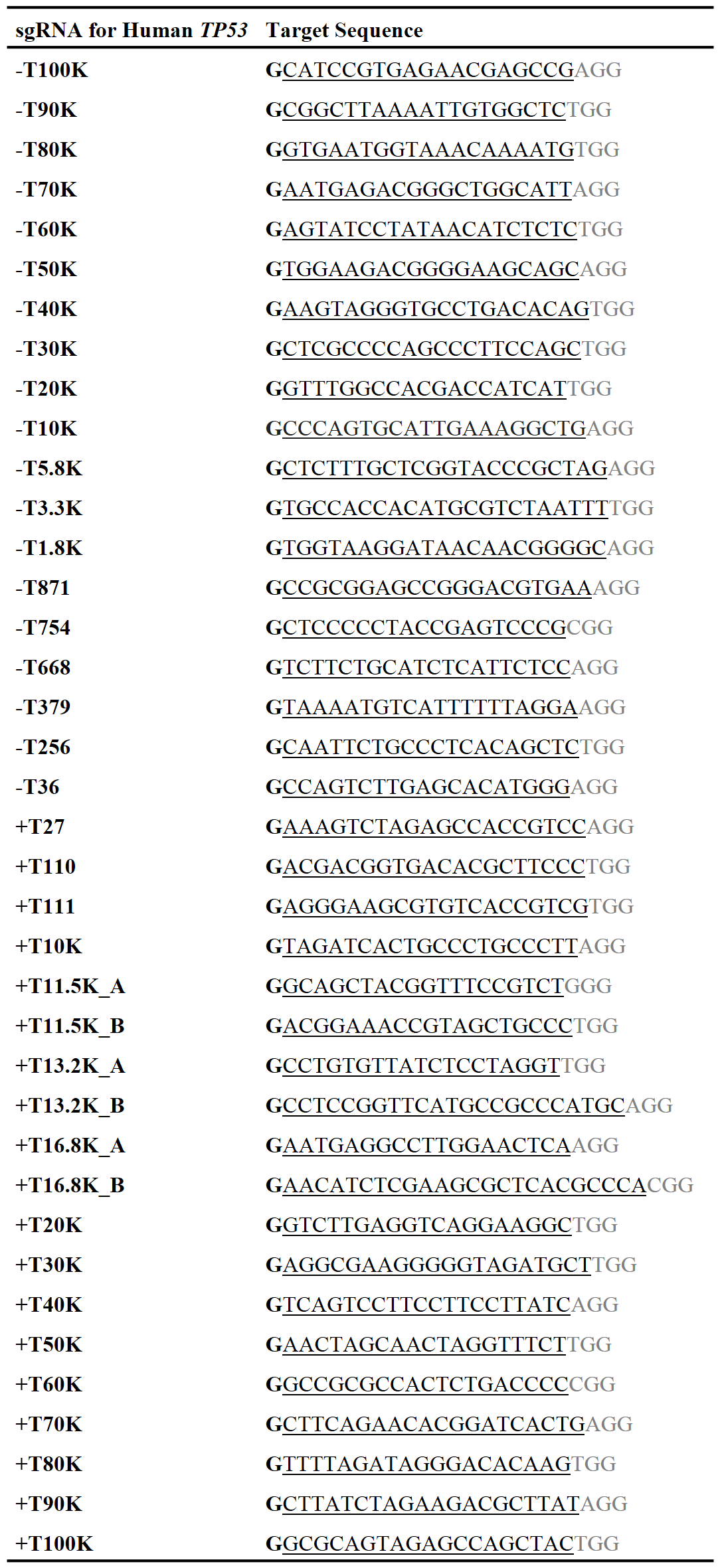

Supplement: Table S1 — Target sequences for sites within and flanking human TP53 . The leading G nucleotide required for U6 promoter expression is in bold. The underlined following 19 to 24 nucleotides comprise the target sequence. The PAM site is in gray. (TIF) [file pone.0113232.s006.tif]

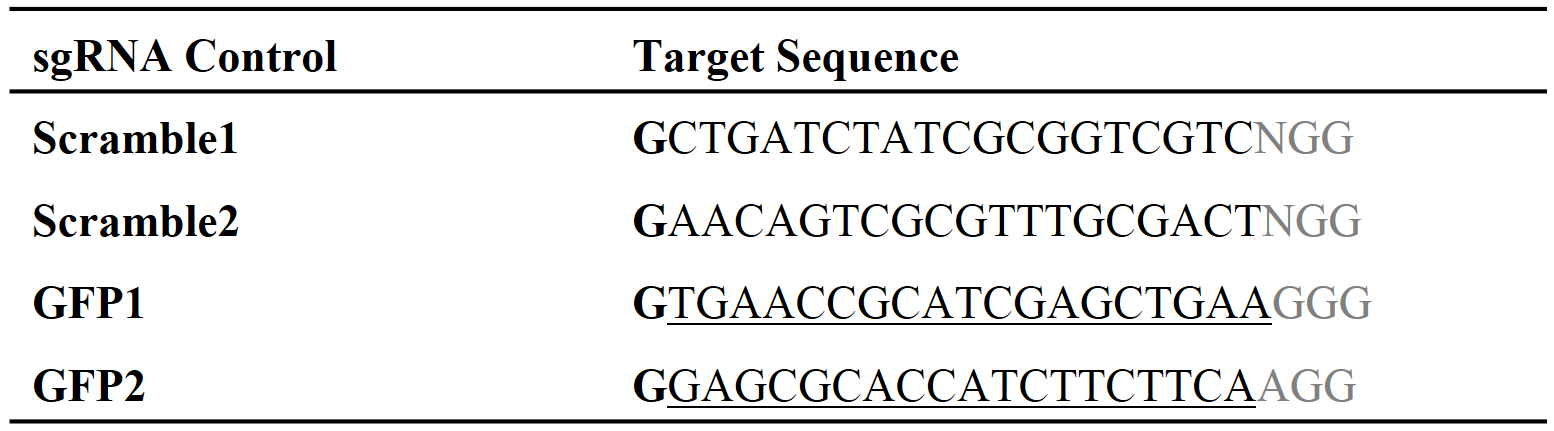

Supplement: Table S2 — Control target sequences. The leading G nucleotide required for U6 promoter expression is in bold. The underlined following 19 to 24 nucleotides comprise the target sequence. The PAM site is in gray. (TIF) [file pone.0113232.s007.tif]

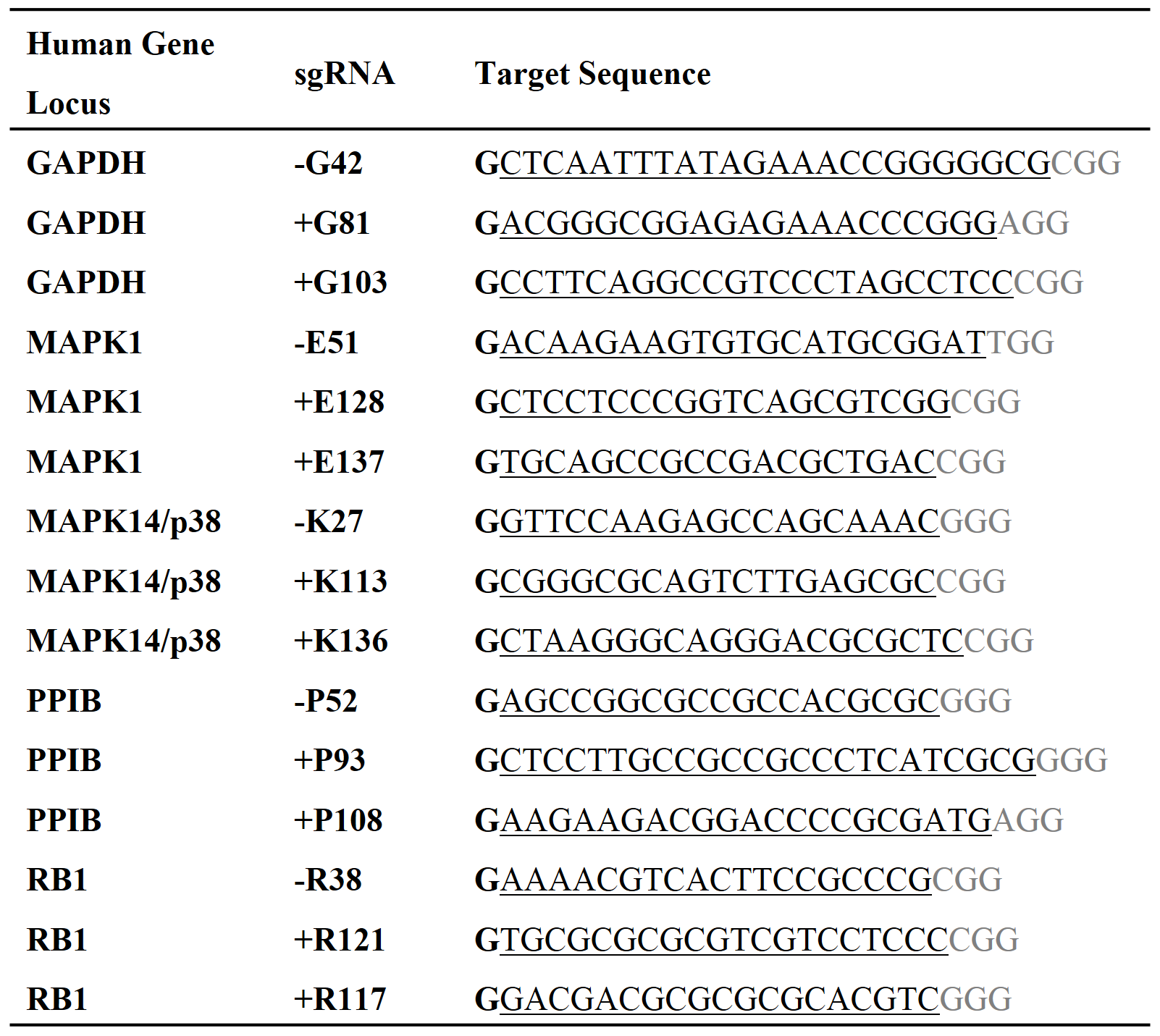

Supplement: Table S3 — Target sequences within the promoter of various human genes of interest. The leading G nucleotide required for U6 promoter expression is in bold. The underlined following 19 to 24 nucleotides comprise the target sequence. The PAM site is in gray. (TIF) [file pone.0113232.s008.tif]

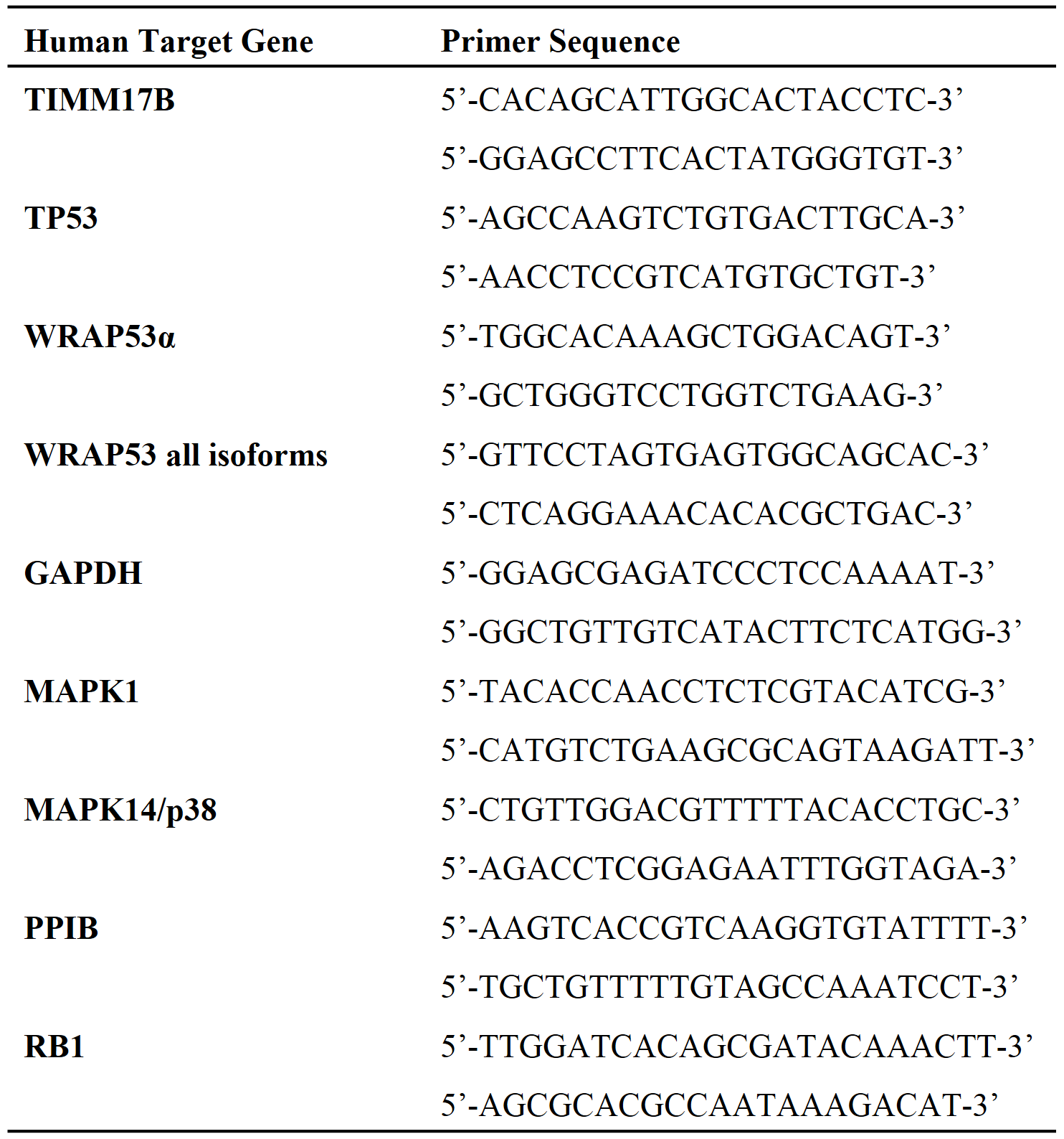

Supplement: Table S4 — Primer sequences used for quantitative RT-PCR. (TIF) [file pone.0113232.s009.tif]
